# Reduced olfactory bulb volume accompanies olfactory dysfunction after mild SARS-CoV-2 infection

**DOI:** 10.1038/s41598-024-64367-z

**Published:** 2024-06-11

**Authors:** Marvin Petersen, Benjamin Becker, Maximilian Schell, Carola Mayer, Felix L. Naegele, Elina Petersen, Raphael Twerenbold, Götz Thomalla, Bastian Cheng, Christian Betz, Anna S. Hoffmann

**Affiliations:** 1 Department of Neurology, University Medical Center Hamburg-Eppendorf, Martinistraße 52, 20246 Hamburg, Germany; 2 Department of Otorhinolaryngology and Head and Neck Surgery, University Medical Center Hamburg-Eppendorf, Hamburg, Germany; 3 Population Health Research Department, University Heart and Vascular Center, Hamburg, Germany; 4 Department of Cardiology, University Heart and Vascular Center, Hamburg, Germany; 5 German Center for Cardiovascular Research (DZHK), Partner Site Hamburg/Kiel/Luebeck, Hamburg, Germany; 6 University Center of Cardiovascular Science, University Heart and Vascular Center, Hamburg, Germany

**Keywords:** Central nervous system infections, Viral infection

## Abstract

Despite its high prevalence, the determinants of smelling impairment in COVID-19 remain not fully understood. In this work, we aimed to examine the association between olfactory bulb volume and the clinical trajectory of COVID-19-related smelling impairment in a large-scale magnetic resonance imaging (MRI) analysis. Data of non-vaccinated COVID-19 convalescents recruited within the framework of the prospective Hamburg City Health Study COVID Program between March and December 2020 were analyzed. At baseline, 233 participants underwent MRI and neuropsychological testing as well as a structured questionnaire for olfactory function. Between March and April 2022, olfactory function was assessed at follow-up including quantitative olfactometric testing with Sniffin’ Sticks. This study included 233 individuals recovered from mainly mild to moderate SARS-CoV-2 infections. Longitudinal assessment demonstrated a declining prevalence of self-reported olfactory dysfunction from 67.1% at acute infection, 21.0% at baseline examination and 17.5% at follow-up. Participants with post-acute self-reported olfactory dysfunction had a significantly lower olfactory bulb volume at baseline than normally smelling individuals. Olfactory bulb volume at baseline predicted olfactometric scores at follow-up. Performance in neuropsychological testing was not significantly associated with the olfactory bulb volume. Our work demonstrates an association of long-term self-reported smelling dysfunction and olfactory bulb integrity in a sample of individuals recovered from mainly mild to moderate COVID-19. Collectively, our results highlight olfactory bulb volume as a surrogate marker that may inform diagnosis and guide rehabilitation strategies in COVID-19.

## Introduction

The coronavirus disease 2019 (COVID-19) pandemic, which is caused by severe acute respiratory syndrome coronavirus 2 (SARS-CoV-2), has affected societies worldwide. Olfactory dysfunction is among the most common symptoms in COVID-19 with a reported prevalence of up to 85%^1–8^. COVID-19 features olfactory dysfunction in varying degrees—e.g., anosmia, hyposmia or parosmia—which occur often before the onset of respiratory symptoms^3^. Compared to other COVID-19-related symptoms like cough, fever or fatigue, olfactory dysfunction proved to be more predictive of SARS-CoV-2 infection^9,10^. Despite its relevance, the understanding of the pathophysiology of SARS-CoV-2-related olfactory dysfunction is still incomplete.

There is ongoing research in the mechanisms of COVID-19-related olfactory dysfunction. Commonly, anosmia in the absence of rhinorrhea or nasal congestion is described as an early symptom which suggests other causal mechanisms than a common cold with conductive deficits causing olfactory dysfunction^3,4,11,12^. SARS-CoV-2 might affect the olfactory system at different breakpoints of its trajectory ranging from disruption of sustentacular cells and olfactory sensory neurons situated in the olfactory mucosa to functional disarray of the olfactory cortex^13,14^. Yet, there is only vague understanding of how these aspects relate to clinical outcomes.

Magnetic resonance imaging (MRI) provides a promising avenue to investigate the pathomechanistic substrates of olfactory dysfunction in SARS-CoV-2 infection in vivo. Previous MRI studies put emphasis on the integrity of the olfactory bulb (OB) as a structural correlate of olfactory function in general^15–17^. Volume reduction of the OB accompanies olfactory loss in conditions like acute or chronic rhinosinusitis and head trauma^18^. Abnormalities in psychophysical olfactory testing are demonstrably associated with OB volume alterations in health and disease^19–21^. Furthermore, the duration and degree of olfactory loss is proportional to the OB volume^22^. So far, studies relating OB volumetry and olfactory function in COVID-19 rely on case reports and small sample sizes yielding heterogeneous results^14,23–27^. Therefore, further investigations are warranted.

Based on previous evidence, we hypothesized that olfactory dysfunction in COVID-19 corresponds with interindividual volumetric differences of the OB. To test our hypothesis, we measured the OB volume using structural MRI, comparing individuals with and without reported smell impairment post-COVID-19, and performed a longitudinal assessment of olfactory function in a large sample of individuals recovered from mainly mild to moderate COVID-19. We also addressed if the OB volume is predictive for the long-term self-reported olfactory function. As affections of the olfactory system might precede COVID-19-related neuropathology, we additionally probed for a relationship of OB alterations and neuropsychological test score results in an exploratory analysis. With this work we aimed to further the understanding of the effects SARS-CoV-2 exerts on the olfactory system and deepen our insight in the longitudinal trajectory of subjective smelling impairment as well as the pathophysiology underlying the clinical sequelae of COVID-19.

## Methods

### Study population and clinical examination

**Figure 1:**
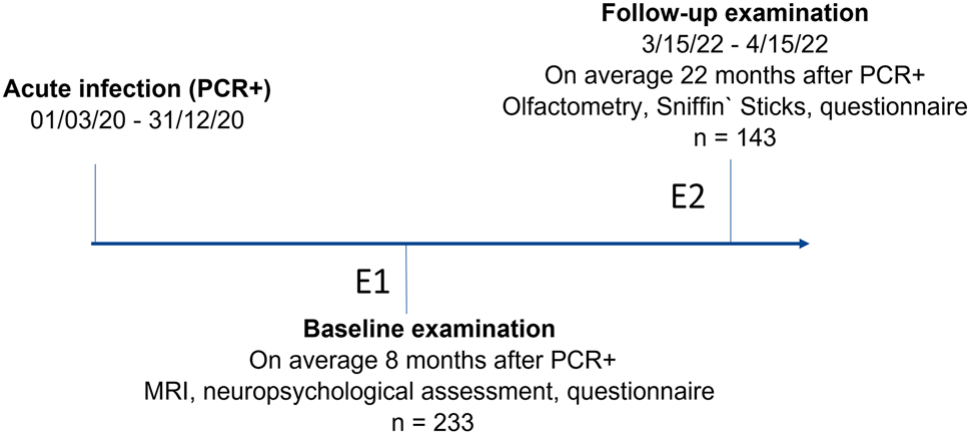
Study timeline. *E1* examination at baseline, *E2* examination at follow-up; *PCR + *  positive polymerase chain reaction test for SARS-CoV-2.

In this work, we investigated data from participants of the Hamburg City Health Study (HCHS) Covid Program with available MRI data. The HCHS is a prospective, single-center cohort study that aims to elucidate risk and prognostic factors in major chronic diseases. A detailed description of the study design has been published separately^28,29^. Our reporting complies with the Strengthening the Reporting of Observational Studies in Epidemiology (STROBE) statement guidelines^30^. In brief, citizens of the city of Hamburg, Germany, were considered for the HCHS COVID Program enrollment if they met two criteria: (1) a laboratory-confirmed positive polymerase chain reaction (PCR) test for SARS-CoV-2, which was obtained between 1st March and 31st December 2020 but at least 4 months prior to study enrollment; (2) age between 45 and 74 years at the time of inclusion. An invitation was issued upon identification via the clinical information system of the University Medical Center Hamburg-Eppendorf or a response to a public call for participation. Recruited participants underwent an extended study protocol of the Hamburg City Health Study (HCHS): besides the standard HCHS work up including MRI and assessment of cognitive function (Trail Making Test B, Word List Recall, Animal Naming Test, Mini Mental State Exam), depressive symptoms (PHQ-9) and quality of life (EQ-5D), participants were required to retrospectively report on disease severity and SARS-CoV-2-associated symptoms via a structured questionnaire^31^. Assessments of the baseline examination could take place at different days depending on individual participant availability. The baseline examination is designated as E1 in figures and tables. A mild to moderate COVID-19 severity was defined as a symptomatic disease course not requiring intensive care unit treatment. The presented study was only conducted based on the post-SARS-CoV-2 cohort—i.e., the matched cohort of control subjects as described previously could not be leveraged as the required high-resolution T2-weighted MRI data were not available^28,32^. To assess the trajectory of olfactory function, participants were reinvited between 15th March and 15th April 2022. The follow-up investigations comprised a structured questionnaire regarding subjective olfactory function as well as olfactometric assessment via Sniffin’ Sticks Screening 12 test by two trained otorhinolaryngologists (B.B., A.S.H.)^33,34^. The test score ranges from 0 to 12 (0–6: anosmia, 7–10: hyposmia, 11–12: normosmia) and is based on normative information derived from more than 1200 patients assessed with Sniffin’ Sticks Screening and olfactive evoked potentials. MRI and neuropsychological testing were not performed during the follow-up. This follow-up examination is referred to as E2 in figures and tables. Eventually, information about self-reported olfactory dysfunction from structured questionnaires—based on a yes/no-question and a visual analogue scale of olfactory dysfunction severity ranging from 0 (no impairment) to 10 (maximal impairment)—was available for three timepoints: (1) during the acute infection, (2) at the baseline investigation and (3) at follow-up, with acute infection smelling information being assessed retrospectively. For a study timeline refer to Fig. 1.

### Ethics approval

Written informed consent was obtained from all participants. The study was approved by the local ethics committee of the Landesärztekammer Hamburg (State of Hamburg Chamber of Medical Practitioners, PV5131) and conducted complying with the Declaration of Helsinki^35^.

### MRI acquisition

High-resolution 3D T2-weighted images were acquired at baseline on a 3T scanner (MAGNETOM^™^Skyra, Siemens Healthineers, Erlangen, Germany) with the following sequence parameters: TR = 3200 ms, TE = 407 ms, 256 axial slices, ST = 0.94 mm, and IPR = 0.9 × 0.9 mm.

### Olfactory bulb segmentation

**Figure 2:**
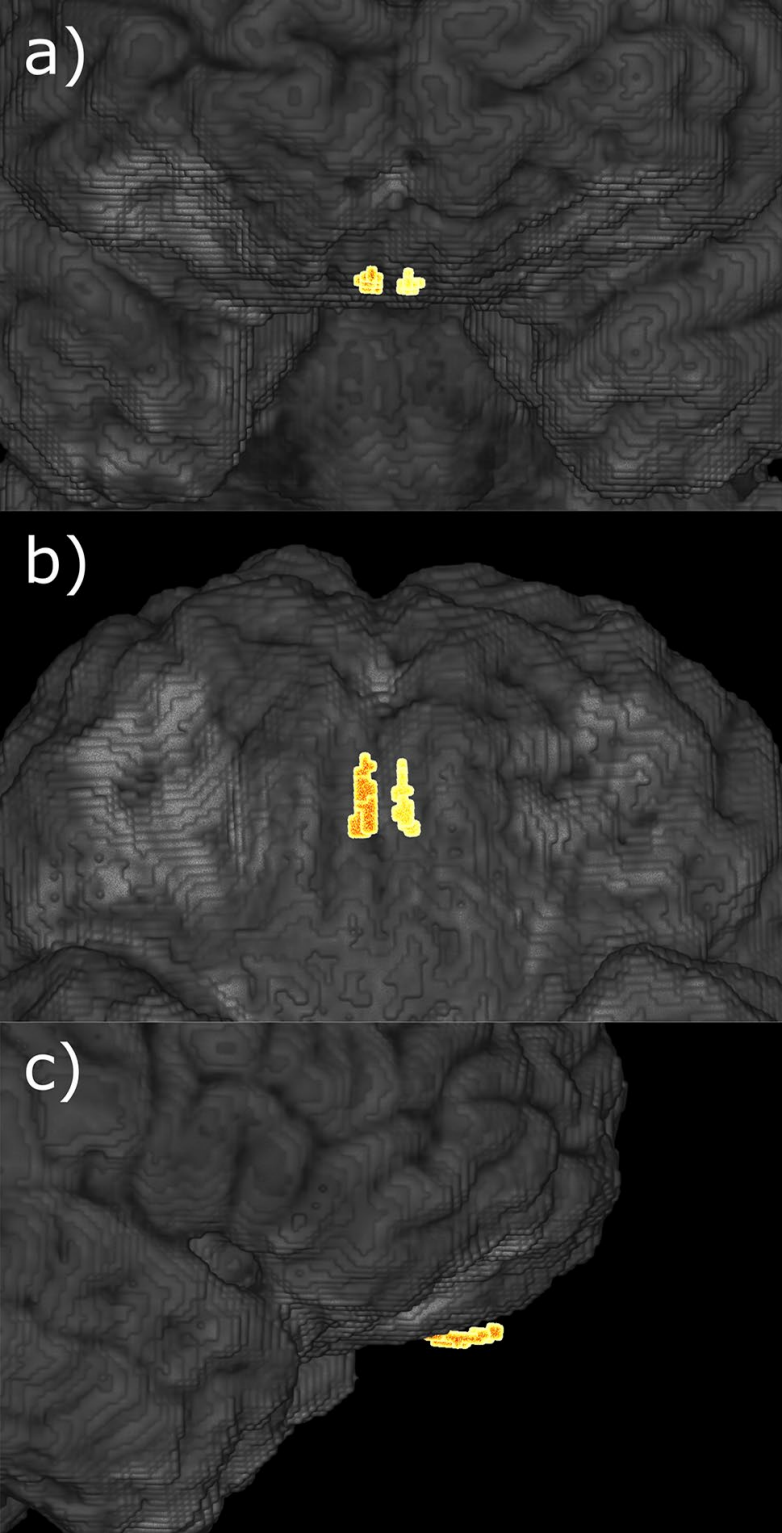
Exemplary 3D visualization of olfactory bulb segmentation results. Volumetric visualization of the left and right olfactory bulb (highlighted) and surrounding brain areas. (**a**) coronal, anterior–posterior; (**b**) axial, inferior-superior; (**c**) sagittal, right-left.

We performed OB segmentation on high-resolution T2-weighted images leveraging a novel fully-automated deep learning-based pipeline specifically designed for OB volumetry^36^. All resulting segmentations underwent visual quality assessment. Exemplary segmentation results are illustrated in Fig. 2 as well as Supplementary Materials S1*-*4. The summed volume of both OBs was used for further analysis.

### Statistical analysis

OB volume and olfactometry scores were compared between individuals with and without self-reported olfactory dysfunction (based on yes/no question) at different timepoints employing analyses of covariance (ANCOVA). Multiple linear regression analysis was performed for assessing the linear relationship of olfactometry scores as well as neuropsychiatric scores with OB volume. A further ANCOVA was performed to test whether the OB volume at baseline differed between individuals with sustained self-reported olfactory dysfunction at follow-up and those that recovered until then. The association between the OB volume and the time interval from positive PCR to examination was assessed via Spearman correlation. Age, sex and smoking behavior were included as covariates in ANCOVAs and linear models as they represent potential confounders^37^. Statistical computations and plotting were performed in Python 3.9.7 harnessing matplotlib (v.3.5.1), numpy (v.1.22.3), pandas (v.1.4.2), pingouin (v.0.5.1) and seaborn (v.0.11.2)^38–42^.

## Results

### Sample characteristics

Data from 233 HCHS Covid Program participants were available for primary analysis. Nine subjects were excluded pre-analysis: 3 since they reported to have had olfactory dysfunction before their SARS-CoV-2 infection, 3 because of a non-detectable OB in T2-weighted MRI and 3 because of erroneous segmentations. Thus, data from n = 224 participants were available for the final analysis. Sample characteristics are summarized in Table 1. On average participants were 55.79 ± 7.25 [mean ± SD] years old, 44.2% were female and 5.4% were current smokers. 8.4% of participants were hospitalized due to COVID-19.

**Table 1:** Characteristics of Post-SARS-CoV-2 Individuals.

Demographics	
Age in years, mean ± SD (n)	55.79 ± 7.25 (224)
Female sex at birth, % (n)	44.2 (224)
Education in years, mean ± SD (n)	15.83 ± 2.52 (224)
Current smokers, % (n)	5.4 (224)
Allergic rhinitis, %	33.3 (224)
Diabetes, %	4.4 (224)
COVID-19	
Days between first positive SARS-CoV-2 PCR test and baseline (E1), mean ± SD (n)	253.15 ± 84.46 (223)
Days between first positive SARS-CoV-2 PCR test and follow-up (E2), mean ± SD (n)	663.51 ± 109.74 (143)
Hospitalization, %	8.5 (224)
Olfaction and olfactory bulb volume	
Self-reported olfactory dysfunction at acute infection, %	67.1 (143)
Self-reported olfactory dysfunction severity (VAS), acute infection, mean ± SD (n)	7.62 ± 2.76 (95)
Self-reported olfactory dysfunction, E1, % (n)	21.0 (143)
Self-reported olfactory dysfunction severity (VAS), E1, ± SD (n)	3.79 ± 1.78 (29)
Self-reported olfactory dysfunction, E2, % (n)	17.5 (143)
Self-reported olfactory dysfunction severity (VAS), E2 mean ± SD (n)	2.96 ± 2.10 (24)
Olfactometry score, E2, mean ± SD (n)	10.18 ± 1.78 (143)
Olfactory bulb volume in mm^3^, E1, mean ± SD (n)	45.53 ± 12.85 (224)
Neuropsychological scores	
Trail Making Test B, E1, mean ± SD (n)	69.96 ± 24.84 (206)
Word List Recall Test, E1, mean ± SD (n)	8.51 ± 1.67 (205)
Animal Naming Test, E1, mean ± SD (n)	28.25 ± 6.05 (206)
Mini Mental State Exam, E1, mean ± SD (n)	28.42 ± 1.24 (205)
Patient Health Questionnaire 9, E1, mean ± SD (n)	3.54 ± 3.69 (218)
EQ5D, E1, mean ± SD (n)	80.74 ± 14.42 (206)

*n* number of data points, *PCR* polymerase chain reaction, *post-SARS-CoV-2* individuals who have recovered from a severe acute respiratory coronavirus type 2 infection, *E1* examination at baseline, *E2* examination at follow-up, *SD* standard deviation.

### Longitudinal trajectory of olfactory function

**Figure 3:**
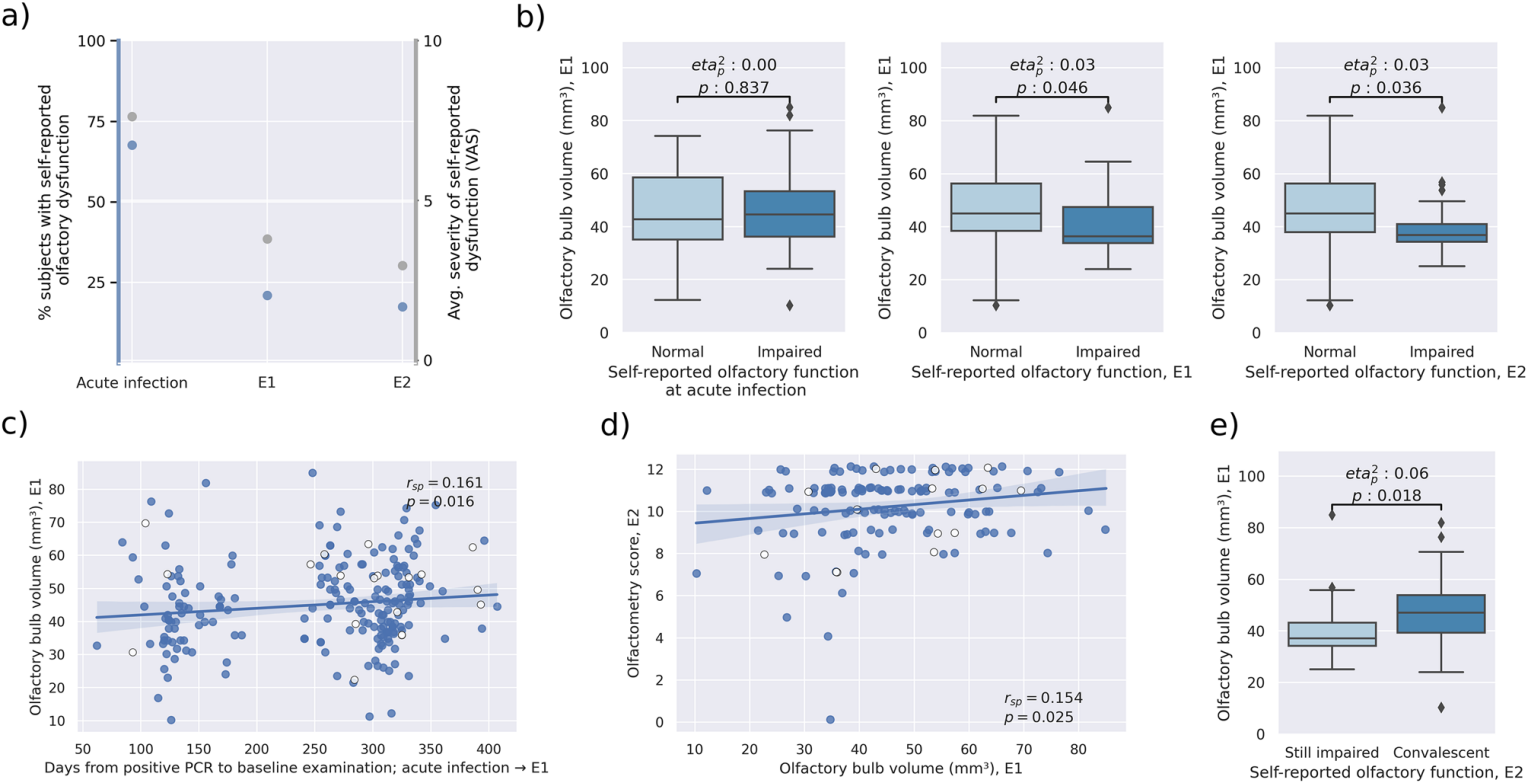
Association of olfactory bulb volume and olfactory function. (**a**) Trajectory of olfactory function along timepoints. Blue dots indicate proportion of individuals with self-reported olfactory dysfunction. Gray dots show the average impairment as operationalized by the visual analogue scale. (**b**) Group differences of olfactory bulb volume at baseline between participants with and without self-reported olfactory dysfunction with respect to different timepoints. Olfactory bulb volume at baseline was significantly lower in individuals that exhibited self-reported olfactory dysfunction during both examination timepoints but not during the acute infection. (**c**) Association of the time interval from positive PCR to examination and olfactory bulb volume. A smaller interval was significantly associated with lower olfactory bulb volume. Non-hospitalized participants are represented by blue dots, hospitalized participants by white dots. (**d**) Linear associations between olfactory bulb volume and olfactometry scores. A low olfactory bulb volume at baseline was significantly associated with a lower olfactometry score at follow-up. Dot colors indicate hospitalization status as in c. (**e**) Group differences of olfactory bulb volume between participants with sustained self-reported olfactory dysfunction at follow-up and those with recovered olfaction to that point. Olfactory bulb volume was significantly lower in participants with sustained self-reported olfactory dysfunction. Abbreviations: ${eta}_{p}^{2}$ = partial eta squared indicating the effect size as provided by the analysis of covariance, *p* p-value, ${r}_{sp}$ Spearman correlation coefficient, *PCR* polymerase chain reaction, *E1* examination at baseline, *E2* examination at follow-up.

The baseline examination happened on average 253 ± 84.46 days after the positive PCR test prompting recruitment, the follow-up at 663 ± 109.74 days. 143 participants were available at follow-up (81 study drop-outs). Of these, 67.1% (n = 96) reported olfactory dysfunction during the acute phase of infection, 21.0% (n = 30) at the baseline examination and 17.5% (n = 25) at follow-up (Fig. 3a). None of the participants who reported olfactory dysfunction at follow-up had indicated normal olfactory function at baseline. Assessment of self-reported olfactory dysfunction severity via the visual analogue scale resulted in a coherent trajectory: 7.62 ± 2.76 (acute infection), 3.79 ± 1.78 (baseline), 2.96 ± 2.10 (follow-up).

### Olfactometry

To assess long term olfactory outcomes, olfactometry with the Sniffin’ Sticks Screening 12 test was performed on participants at follow-up. Olfactometry scores at follow-up were on average 10.18 ± 1.78. Participants who self-reported olfactory dysfunction during acute infection showed no significant difference in olfactometry scores at follow-up compared to those reporting normal smelling during the acute infection (mean ± SD, 10.04 ± 1.97 vs. 10.46 ± 1.31, ${eta}_{p}^{2}$=0.01, *p* = 0.211; Supplementary Materials S5). Yet, participants with self-reported olfactory dysfunction at baseline had lower olfactometric scores at follow-up than those reporting normal smelling at baseline (mean ± SD, 8.67 ± 2.61 vs. 10.58 ± 1.22, ${eta}_{p}^{2}$=0.19, *p* < 0.005) and the same applied for participants that reported to be impaired at follow-up compared to those reporting normal smelling at baseline (mean ± SD, 8.40 ± 2.74 vs. 10.56 ± 1.22, ${eta}_{p}^{2}$=0.22, *p* < 0.005).

### Olfactory bulb volume and olfactory function

The mean OB volume was 45.53 ± 12.85 mm^3^. The ANCOVA yielded no significant group difference in OB volume between individuals with and without self-reported olfactory dysfunction at the acute infection (mean ± SD, 45.85 ± 12.92 vs. 45.21 ± 15.16, ${eta}_{p}^{2}$=0.00, *p* = 0.837; Fig. 3b). Individuals with self-reported olfactory dysfunction at baseline and at follow-up had significantly lower OB volume at baseline than subjects reporting normal smelling at the respective timepoints (mean ± SD, baseline: 40.76 ± 13.08 vs. 46.74 ± 13.66, ${eta}_{p}^{2}$=0.03, *p* = 0.046; follow-up: 40.45 ± 12.59 vs. 46.55 ± 13.76, ${eta}_{p}^{2}$=0.03, *p* = 0.036). In participants with self-reported olfactory dysfunction lower OB volume was accompanied by a shorter time-period between a positive PCR for SARS-CoV-2, signifying the timepoint of acute SARS-CoV-2 infection, and the baseline examination (${r}_{sp}$=0.161, *p* = 0.016; Fig. 3c).

### Longitudinal prediction of olfactory function

OB volume derived from MRI at baseline was significantly linearly associated with olfactometry scoring at follow-up (${r}_{sp}$=0.154, *p* = 0.025; Fig. 3d). This result remained robust if the participant with an olfactometry score of 0 was excluded as an outlier (Supplementary Materials S6). Participants with sustained self-reported olfactory dysfunction at follow-up had lower OB volume at baseline (mean ± SD, 40.64 ± 12.83 vs. 47.58 ± 12.56, ${eta}_{p}^{2}$=0.06, *p* = 0.018; Fig. 3e) than those that reported recovery of normal smelling by the time of reassessment.

### Clinical analysis

To further explore potential clinical implications of our findings, correlations of OB volume and neuropsychological cognitive test scores and psychiatric test scores were performed. To summarize, no significant associations of OB volume and scores of the Trail Making Test B (${r}_{sp}$=0.014, *p* = 0.722), Animal Naming Test (${r}_{sp}$=− 0.090, *p* = 0.069), Word List Recall Test (${r}_{sp}$=-0.050, *p* = 0.587), Mini Mental State Exam (${r}_{sp}$=− 0.025, *p* = 0.689), PHQ-9 (${r}_{sp}$=0.098, *p* = 0.404) and EQ-5D (${r}_{sp}$=− 0.009, *p* = 0.306) were found. Corresponding visualizations are displayed in Supplementary figure S7.

## Discussion

We report on an association of OB volume and self-reported as well as measured olfactory function in a large sample of mainly mildly to moderately affected COVID-19 convalescents. Longitudinal assessment demonstrated sustained self-reported olfactory dysfunction up to two years after acute infection. Participants suffering from subjective olfactory dysfunction beyond acute infection had a significantly lower OB volume at baseline than individuals reporting normal smelling at that timepoint. Moreover, OB volume was predictive for olfactometric performance in the Sniffin’ Sticks test 22 months after the acute infection as well as for the binary outcome of self-reported recovery from olfactory dysfunction at follow-up. Neuropsychological test performances were not significantly associated with OB volume. Taken together, our findings suggest that lower volume of the OB may be a promising surrogate marker of smelling function in COVID-19 at post-acute disease stages.

### Longitudinal trajectory of olfactory dysfunction

Its frequency and the concomitant effects on quality of life render olfactory dysfunction a burdensome symptom of COVID-19. Our 2 year longitudinal assessment of olfactory function provided insights about time-dependent development of these symptoms. At acute infection, the proportion of participants reporting olfactory dysfunction was 67.1% which is coherent with previous study reports ranging from 30 to 85%^1–4,6,7^. After the acute infection, the prevalence decreased to 21% at the baseline examination (on average 253 days post infection) and 17.5% at follow-up (on average 664 days post infection; Fig. 3a). These numbers support previous literature finding high rates of long-term olfactory dysfunction in COVID-19^43–45^. Of note, a recent meta-analysis reports persistent olfactory dysfunction in less patients (11.6%; 95% confidence interval 5.2% to 23.9%)^46^. We attribute this difference to design differences with respect to our work: the studies included in the meta-analysis also considered young adults (> 18 years) possibly exhibiting higher regenerative capacity as well as recent SARS-CoV-2 variants demonstrably affecting patient olfaction less severely than the wild type variant mainly prevalent at the pandemic onset^47^. Overall, these findings indicate that although most former COVID-19 patients completely recover, subjective olfactory dysfunction persisted in a relevant proportion of individuals.

To assess long term olfactory outcomes, olfactometry with the Sniffin’ Sticks Screening 12 test was performed at follow-up. Participants that reported olfactory dysfunction beyond the acute infection, plausibly showed significantly lower olfactometry scores than individuals reporting normal smelling or recovering subjects. Collectively, our results support previous evidence of sustained olfactory dysfunction in around 20% of patients underscoring the possible long lasting burden following a SARS-CoV-2 infection^6,44^.

### Long-term olfactory dysfunction is associated with lower olfactory bulb volume

Olfactory dysfunction is demonstrably accompanied by lower OB volume in many otorhinolaryngological conditions like post-infectious olfactory disorder, head trauma as well as acute and chronic rhinosinusitis^18^. In line with this notion, our work showed a lower OB volume in individuals with sustained self-reported olfactory dysfunction after the acute SARS-CoV-2 infection indicating COVID-19-related OB atrophy (Fig. 3b). Thus, our results corroborate previous reports derived from case studies and small samples suggesting lower OB volume in SARS-CoV-2-induced olfactory dysfunction^23–26,48^.

Previous reports demonstrated an inverse correlation between OB volume and the duration of symptoms in post-infectious olfactory disorder suggesting its predictive capacity^22^. To further investigate this, we related the OB volume with olfactometry scores which were acquired approximately 1 year after the MRI. Notably, the OB volume predicted olfactometry scores at follow-up (Fig. 3d). Yet, the observed correspondence was of a rather low degree, indicating that further determinants of long-term olfactory dysfunction should be considered. Possibly, MRI assessment closer to the acute infection would have resulted in the observation of stronger effects. Furthermore, OB volume was higher in participants in which reported a fully recovered smelling sense until follow-up compared to those reporting sustained olfactory dysfunction (Fig. 3e). Hence, the OB volume appears to be a predictor of recovery from olfactory dysfunction, i.e., potentially capturing the severity of damage SARS-CoV-2 exerts on the olfactory system. Interestingly, individuals exhibited higher OB volume the longer the time interval between the positive PCR and the MRI was (Fig. 3c). This might indicate that an increasing OB volume may reflect recovery of olfactory function. However, longitudinal imaging assessment is warranted here. Taken together, these findings suggest that a lower OB volume indicates more severe disruption of the olfactory system and predict persistent olfactory dysfunction in COVID-19 patients.

### Pathomechanistic underpinnings of abnormal olfactory bulb volume

There are multiple potential mechanisms that might explain the observed link between self-reported olfactory dysfunction and OB dysintegrity. In the olfactory mucosa, olfactory sensory neurons (OSN)—sensing molecular signatures as odor information—as well as supporting epithelial cells (sustentacular cells) ensure proper sense of smell. Sustentacular cells express angiotensin-converting enzyme 2 (ACE-2) and appear to be a major infection target of the virus^13,49^. As they support OSN in a glial-like fashion, impairment of sustentacular cells is considered to contribute to COVID-19-related olfactory dysfunction^50^. How OSN are affected by SARS-CoV-2 is controversial. Discussed mechanisms are neurotropism, affection by an impaired support system and damage caused by the immune response to the virus^13,50,51^. Recent analyses failed to detect signs of neurotropism and neural invasion through SARS-CoV-2 challenging the notion of the olfactory system serving the virus as an entry point^50,52,53^. As the OB serves as a relay for projections from the OSN, volumetric reductions might occur as an effect of indirect OSN affection—e.g., via inflammation or microvasculopathy—rather than direct damage from the virus leading to reduced tissue integrity^53^. Taking the link between OB integrity and long-term olfactory function into account, OB volume might serve as an indicator of severe structural disruption of the olfactory system which corresponds with unfavorable outcomes. Nonetheless, further longitudinal neuroimaging research is warranted to support this notion.

### Olfactory bulb volume is not associated with neuropsychological test scores

By now, COVID-19 is recognized to cause post-acute neurological and psychiatric symptoms like executive dysfunction, fatigue, anxiety, depression and sleep impairment^54–57^. Coherent with these observations, a comprehensive MRI analysis on COVID-19 convalescents from the UK Biobank has shown widespread gray matter volume reductions in areas receiving projections from the olfactory cortex^55^. Its evident exposition to deleterious SARS-CoV-2 effects renders the OB a candidate indicator of COVID-19-related neuropathology beyond the olfactory system. Thus, we tested whether the OB volume is associated with results of neuropsychological test scores. OB volume showed no significant association with tests of cognitive function (Trail Making Test B, Word List Recall, Animal Naming Test), depressive symptoms (Patient Health Questionnaire-9, PHQ-9) and quality of life (EQ-5D). Consequently, pathology of the olfactory system might be disjunct to non-olfaction-related neuropathology in SARS-CoV-2 infection. Nonetheless, our findings might be partially attributable to the overall mild to moderate disease course captured in our sample resulting in negative results. Further investigations of determinants of neurological and psychiatric sequelae of COVID-19 are necessary.

### Strengths and limitations

The strengths of this work lie in its considerable sample size; high quality imaging and phenotypical data; a modern fully-automated MRI-based segmentation of the OB enabling volumetry at scale; olfactory assessment at different time points post infection, including quantitative olfactory testing (at follow-up) providing longitudinal information for up to 2 years. Furthermore, as vaccinations were not yet available at the time of the initial recruitment and baseline examination, their confounding effects are reduced in our investigation. Yet, this study has some limitations. First, MRI acquisition and olfactometry with Sniffin’ sticks were performed only at one timepoint, which makes it difficult to completely address pre-infectious group differences. For instance, our results could partially be explained by individuals with lower OB volume being more susceptible to olfactory dysfunction caused by SARS-CoV-2 infection. Previous work hypothesizes that smaller OB volume and pre-existing reduced number of olfactory receptor neurons increased the patient’s vulnerability to develop post-infectious olfactory loss. With less functional tissue existing in the first place, damage to existing sensory cells might lead to more pronounced olfactory dysfunction^58^. Here, evidence from future longitudinal studies is required. As more severely impaired individuals might be more motivated and thus more likely to participate in our study than the average population, our results could possibly be influenced by our recruitment strategy. Additionally, a more in-depth assessment of olfactory function based on odor thresholds was not conducted at follow-up because high dropout rates due to compliance issues were expected. Parosmia may serve as a potential confounding factor in the observed associations, thus necessitating cautious interpretation of our results. Unfortunately, information on parosmia was not available for this study. Moreover, we cannot fully rule out that the prevalence of subjective olfactory dysfunction over time can be partially attributed to the general population’s baseline, independent of SARS-CoV-2 infection. Measures to mitigate this confounding were to exclude participants that reported to have olfactory dysfunction before COVID-19 as well as asking in a questionnaire specific to COVID-19 symptoms whether they still experience olfactory dysfunction. Lastly, the different SARS-CoV-2 strains appear to differ in terms of olfactory dysfunction frequency and intensity. Our study lacks information about SARS-CoV-2 strains rendering it incapable to address inter-strain differences. However, the initial recruitment for the HCHS COVID Program happened at an early stage of the pandemic most likely addressing the problem of different COVID-19 strains.

## Conclusion

In this work, we performed OB volumetry as a neuroimaging marker of olfactory dysfunction in patients recovered from mainly mild to moderate COVID-19. By revealing an alteration of the OB in participants with self-reported olfactory dysfunction, our results highlight the relevance of the olfactory system in the overall pathophysiology of the disease. However, a connection between OB volume and neuropsychological signs of COVID-19 could not be established. Collectively, these results demonstrate that the OB is a promising target for assessment of olfactory dysfunction in COVID-19.

### Supplementary Information

Supplementary Information.

## Data Availability

Personalized data from individual participants of the HCHS COVID Program is not publicly available due to data protection regulations, but anonymized data can be accessed by interested researchers via a request to the HCHS steering committee based on a material transfer agreement (MTA). The analysis code is publicly available on GitHub (https://github.com/csi-hamburg/2023_petersen_ob_postcovid).
