# Supplementary material for: Reduced olfactory bulb volume accompanies olfactory dysfunction after mild SARS-CoV-2 infection
Source: Sci Rep. 2024 Jun 11;14:13396. doi: 10.1038/s41598-024-64367-z (PMC11167024; doi:10.1038/s41598-024-64367-z)
Supplement: Supplementary file 1 — Supplementary Information. [file 41598_2024_64367_MOESM1_ESM.pdf]

Supplementary Materials

–

Reduced Olfactory Bulb Volume Accompanies Olfactory Dysfunction After Mild SARS-CoV-2 Infection

Marvin Petersen, MD[1#]; Benjamin Becker, MD[2#]; Maximilian Schell[1], Carola Mayer[1], Felix L. Naegele[1], Elina Petersen, MSc[3,4]; Raphael Twerenbold, MD[3,4,5,6]; Götz Thomalla, MD[1]; Bastian Cheng, MD[1]; Christian Betz, MD[2], Anna S. Hoffmann, MD[2]

[1] Department of Neurology, University Medical Center Hamburg-Eppendorf, Hamburg, Germany

[2] Department of Otorhinolaryngology and Head and Neck Surgery, University Medical Center Hamburg-Eppendorf, Hamburg, Germany

[3] Population Health Research Department, University Heart and Vascular Center, Hamburg, Germany

[4] Department of Cardiology, University Heart and Vascular Center, Hamburg, Germany

[5] German Center for Cardiovascular Research (DZHK), partner site Hamburg/Kiel/Luebeck, Hamburg, Germany

[6] University Center of Cardiovascular Science, University Heart and Vascular Center, Hamburg, Germany

[#] These authors contributed equally

S1 - Exemplary visualizations of the olfactory bulb – First subject (1)

First subject – no subjective olfactory dysfunction, male, age range 56-60 y., olfactory bulb volume: 67.68 mm³

Overview of the segmentations of the olfactory bulb. Panel from left to right: coronal, axial, sagittal slices (blue: left olfactory bulb, red: right olfactory bulb)

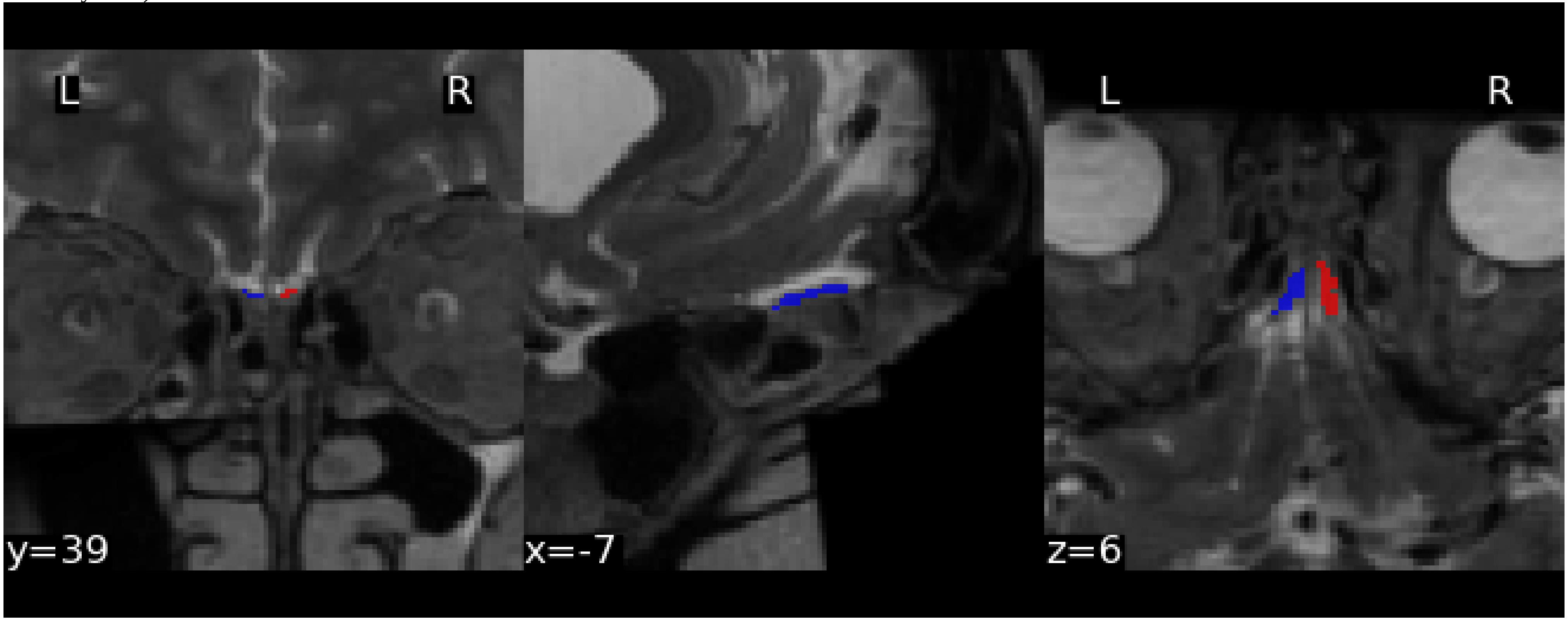

S2 - Exemplary visualizations of the olfactory bulb – First subject (2)

Coronal slices in lightbox view. Left panel without segmentations, right panel with segmentations

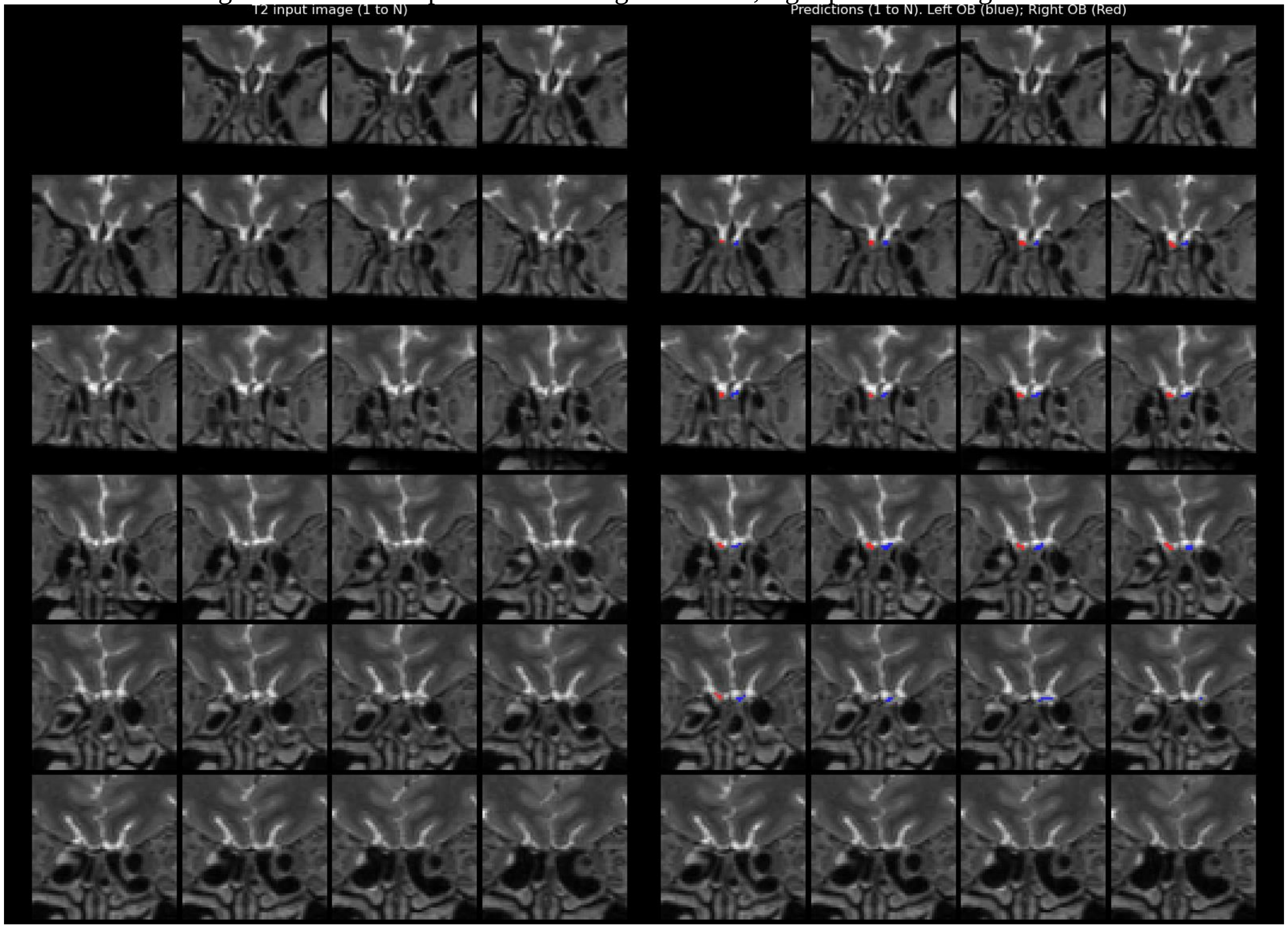

S3 - Exemplary visualizations of the olfactory bulb – Second subject (1)

Second subject – with subjective olfactory dysfunction, male, age range 56-60 years, olfactory bulb volume: 28.67 mm³

Overview of the segmentations of the olfactory bulb. Panel from left to right: coronal, axial, sagittal slices (blue: left olfactory bulb, red: right olfactory bulb)

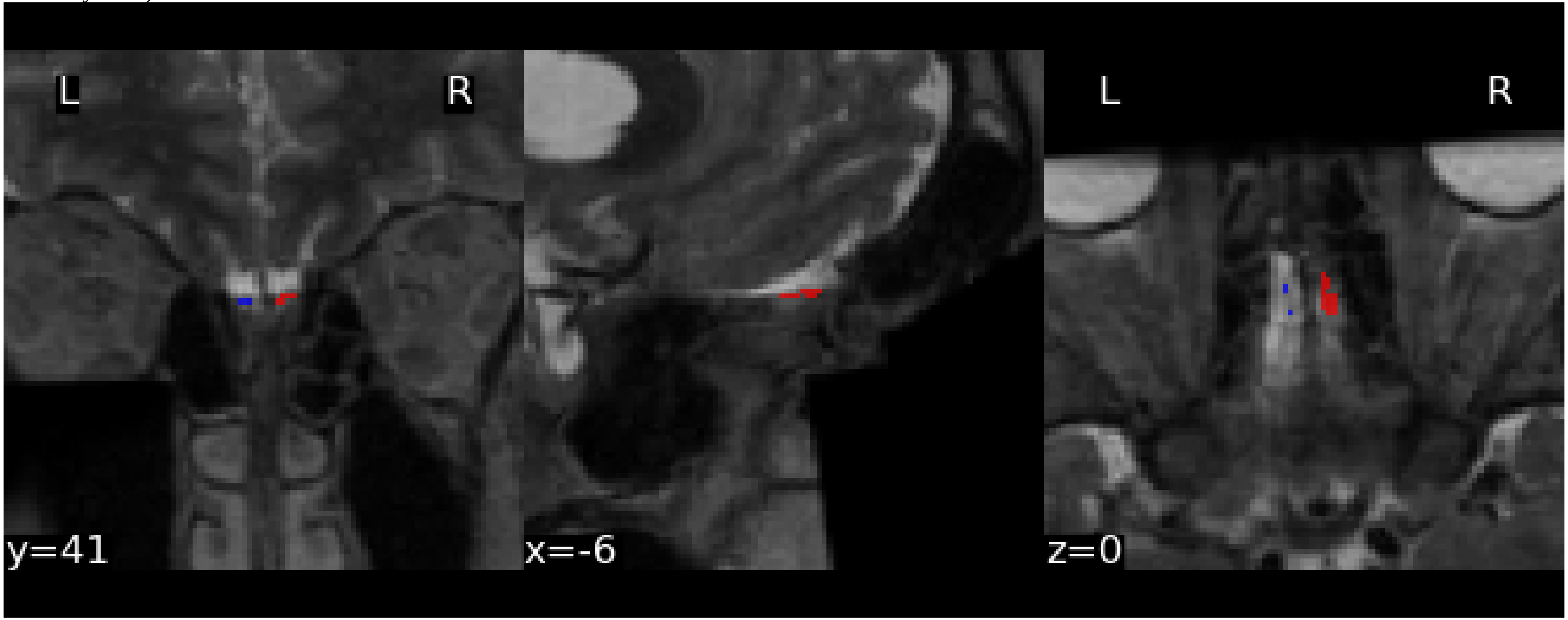

S4 - Exemplary visualizations of the olfactory bulb – Second subject (2)

Coronal slices in lightbox view. Left panel without segmentations, right panel with segmentations

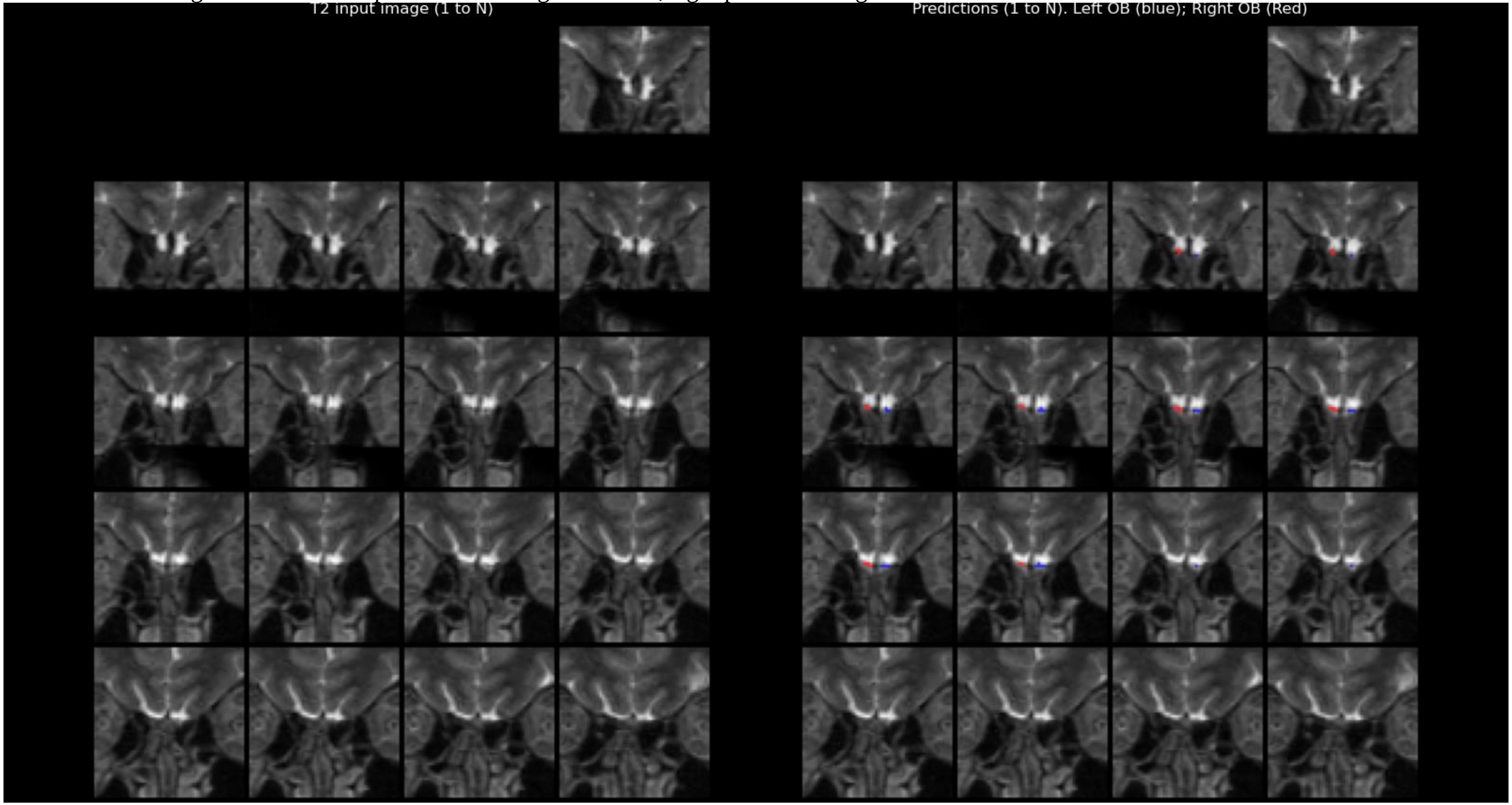

S5 – Group comparison olfactometry scores

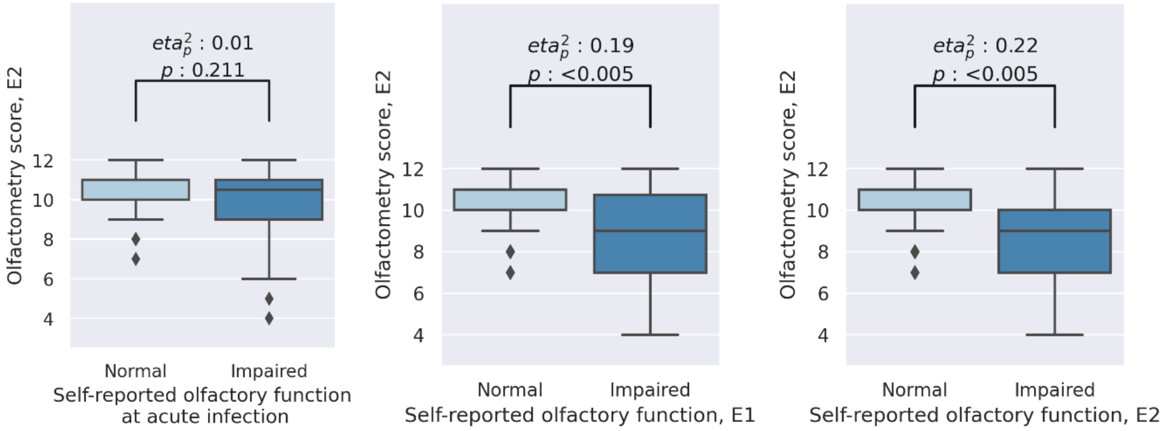

Group differences of olfactometry scores at follow-up between smelling impaired and non-impaired participants at different timepoints. Olfactometry scores at follow-up were significantly lower in individuals that were smelling impaired during both examination timepoints but not during the acute infection (acute infection – mean ± SD, impaired: 10.04 ± 1.97 vs. normal: 10.46 ± 1.31, eta_p^2=0.01, p=0.211; baseline – impaired: 8.67 ± 2.62 vs. normal: 10.58 ± 1.22, eta_p^2=0.19, p<0.005; follow-up – impaired: 8.40 ± 2.74 vs. normal: 10.56 ± 1.22, eta_p^2=0.22, p<0.005).

Abbreviations: eta_p^2 = partial eta squared indicating the effect size as provided by the analysis of covariance, p = p-value, E1 = examination at baseline, E2 = examination at follow-up.

S6 – Linear associations of olfactory bulb volume and olfactometry score after outlier exclusion

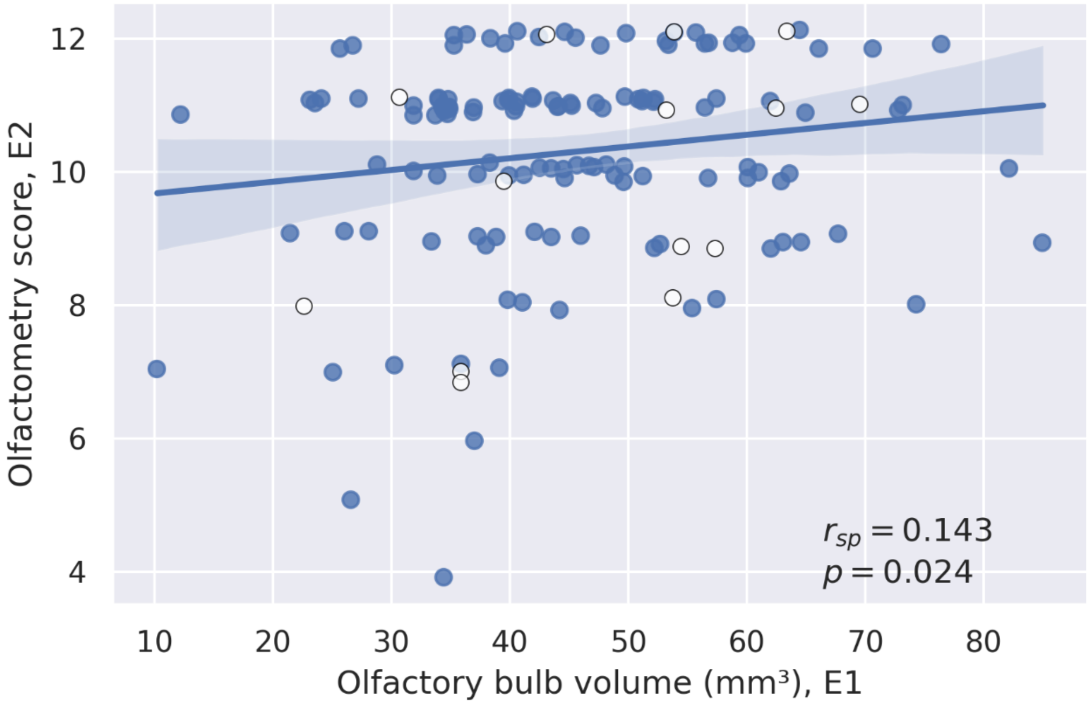

Linear associations between olfactory bulb volume and olfactometry scores after excluding the outlier with an olfactometry score of 0. A low olfactory bulb volume at baseline was significantly associated with a lower olfactometry score at follow-up. Non-hospitalized participants are represented by blue dots, hospitalized participants by white dots.

S7 – Linear associations olfactory bulb volume and neuropsychological test performances

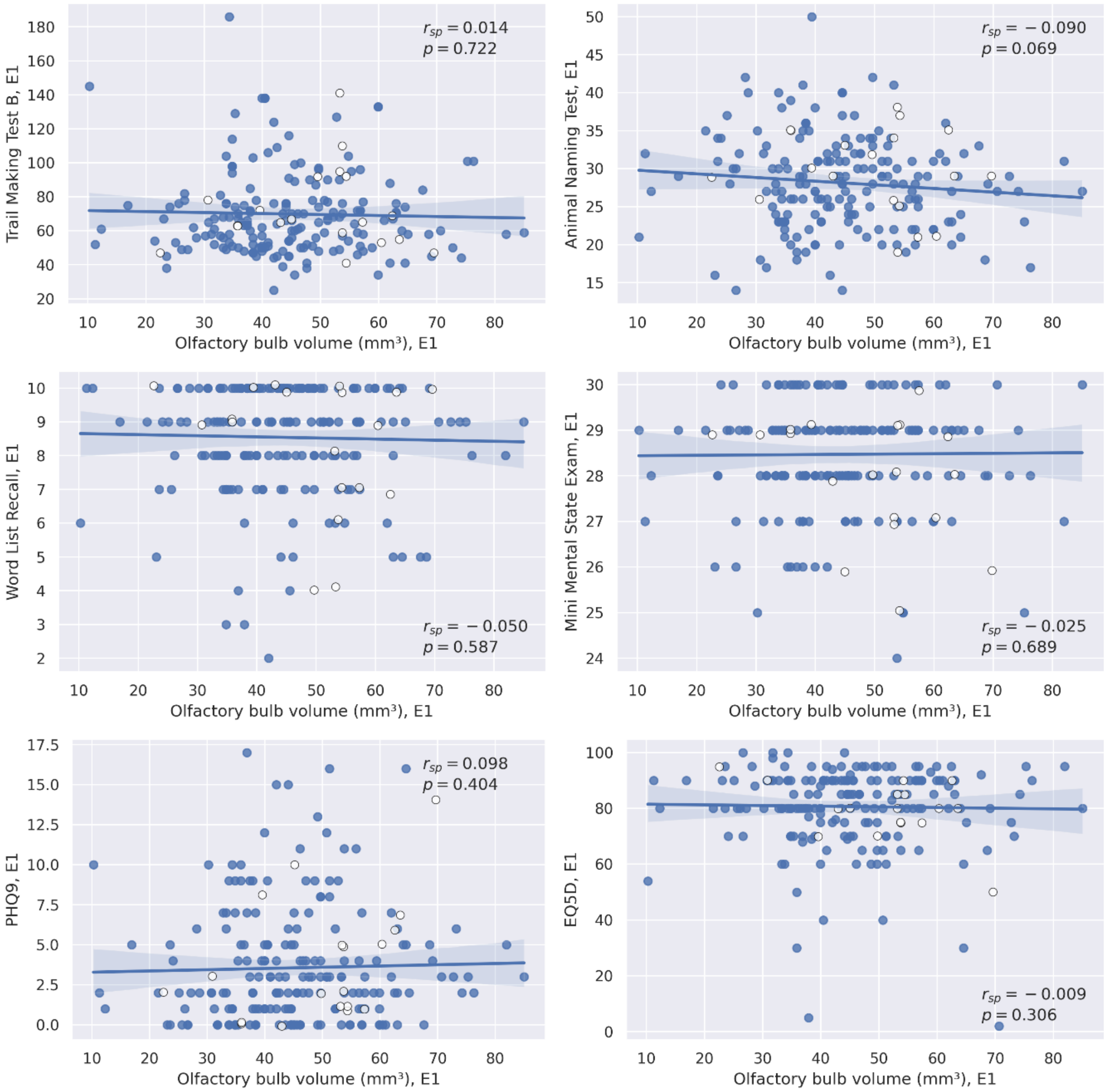

Linear associations between olfactory bulb volume and the Trail Making Test B, Word List Recall, Animal Naming Test, Mini Mental State Exam, Patient Health Questionnaire 9 and EQ-5D are shown. No significant relationships were found. Non-hospitalized participants are represented by blue dots, hospitalized participants by white dots. Abbreviations: p = p-value, PHQ-9 = Patient Health Questionnaire 9, r_{sp} = spearman correlation coefficient, E1 = examination at baseline, E2 = examination at follow-up.
